# Antibiotic treatment for 7 days versus 14 days in patients with uncomplicated bloodstream infections: a Systematic review and meta-analysis of randomized controlled trials and trial sequential analysis

**DOI:** 10.3389/fmed.2025.1617328

**Published:** 2025-08-04

**Authors:** Changyun Zhao, Changqin Chen, Difan Lu, Kailun Cai, Weihang Hu, Wenchao Mao

**Affiliations:** ^1^Department of Critical Care Medicine, Zhejiang Hospital, Hangzhou, China; ^2^Cardiovascular Ultrasound Center of the First Affiliated Hospital, College of Medicine, Zhejiang University, Hangzhou, China

**Keywords:** bloodstream infections, antibiotics, treatment duration, meta-analysis, TSA

## Abstract

**Background:**

The optimal duration of antibiotic therapy for bloodstream infections (BSI) remains a topic of ongoing debate. To address this, we conducted a meta-analysis to evaluate the efficacy and safety of 7-day and 14-day antibiotic regimens in the treatment of BSI.

**Methods:**

We performed a comprehensive search of PubMed, Web of Science, Embase, and the Cochrane Library from the inception of these databases up to March 10th, 2025. Randomized controlled trials (RCTs) comparing 7-day and 14-day antibiotic regimens for the treatment of BSI will be included. The Cochrane risk of bias assessment tool was used to evaluate the risk of bias. The primary outcomes were all-cause mortality, 90-day mortality, while secondary outcomes included relapsed bacteremia, readmissions or prolongation of hospitalization, suppurative complications, emergence of resistance, length of stay in hospital, and adverse events. Trial sequential analysis (TSA) was then conducted.

**Results:**

The meta-analysis included four RCTs involving 4,794 patients. The results indicated no statistically significant differences between the 7-day and 14-day antibiotic regimens in terms of all-cause mortality (RR = 0.96, 95% CI: 0.73–1.25, *p* = 0.75) or 90-day mortality (RR = 0.94, 95% CI: 0.80–1.10, *p* = 0.45). When the analysis was restricted to BSI caused by Gram-negative bacteria (GNB), no statistically significant differences were observed in all-cause mortality or 90-day mortality. The 7-day antibiotic regimen was associated with a significantly shorter length of stay in hospital compared to the 14-day regimen. However, no significant differences were observed in other secondary outcomes or adverse events, including acute kidney injury (AKI), *Clostridioides difficile* infection (CDI), diarrhea, and rash. And the TSA suggested that the current findings may have yielded a false negative conclusion.

**Conclusion:**

For BSI, the 7-day antibiotic regimen was associated with a significantly shorter length of stay in hospital compared to the 14-day regimen, while demonstrated comparable efficacy and safety outcomes. From this perspective, a 7-day antibiotic regimen seems to be more advisable. However, it is imperative to conduct additional large-scale RCTs to validate and substantiate our findings.

**Systematic review registration:**

Registration ID: CRD42024617359; https://www.crd.york.ac.uk/PROSPERO/view/CRD42024617359.

## Introduction

In recent decades, significant progress in medical science, particularly in critical care medicine, has markedly improved patient outcomes through novel theories and technologies. However, bloodstream infections (BSIs), especially those caused by Gram-negative bacteria (GNB), remain a formidable challenge due to their high incidence and mortality rates, imposing substantial healthcare and economic burdens globally (1–3). Recent epidemiological data reveal an upward trend in BSI incidence (4). Concurrently, the pathogen distribution has shifted: while *methicillin-resistant Staphylococcus aureus* (MRSA) prevalence has declined, multidrug-resistant Gram-negative bacteria (MDR-GNB) has become increasingly predominant. Currently, *Escherichia coli* is the leading BSI pathogen, with GNB accounting for over 50% of cases (4, 5). BSI-associated mortality remains alarmingly high, ranging from 10 to 20%, and exceeding 30% in elderly populations (4). These trends underscore the urgent need for optimized therapeutic strategies, enhanced diagnostic approaches, and robust infection control measures to mitigate the impact of BSIs.

The implementation of rational and effective antibiotic therapy is a critical strategy for improving the prognosis of patients diagnosed with BSI. Current research indicated that timely and appropriate antibiotic administration significantly enhances outcomes for patients with BSI and sepsis (6, 7). While the importance of early antibiotic initiation is well-established, the optimal duration of antibiotic therapy remains a subject of ongoing debate. For BSI, specifically short-term central venous catheter-related bloodstream infections (CRBSI), the Infectious Diseases Society of America (IDSA) (8) and the Spanish Society of Clinical Microbiology and Infectious Diseases (SEIMC) (9) provided expert consensus recommendations. According to these guidelines, following the removal of the central venous catheter (CVC), a treatment duration of 7 to 14 days was recommended for uncomplicated infections caused by GNB and *enterococci* (8, 9). However, the optimal duration of antimicrobial therapy for non-CRBSI remains unclear, as no definitive consensus exists. In summary, there is currently insufficient robust evidence to establish the optimal anti-infective treatment regimen for BSI. It is important to recognize that while appropriate antibiotic therapy improves patient outcomes, prolonged antibiotic exposure can increase the risk of adverse drug reactions and contribute to the development of antibiotic resistance (10, 11). Therefore, minimizing the duration of antibiotic therapy is essential to reduce the risk of resistance, decrease the incidence of adverse events, and alleviate the financial burden on healthcare systems.

Several prior meta-analyses have compared the efficacy and safety of long-course antibiotic treatment (>10 days) versus short-course treatment (≤10 days) for BSI. These studies have revealed that short-term and long-term treatment strategies yield similar clinical efficacy, and no statistically significant differences in adverse event rates were detected between the two approaches (12, 13). Another meta-analysis focusing specifically on Gram-negative bacteria bloodstream infections (GNB-BSI) demonstrated comparable efficacy and safety profiles between 7-day and 14-day antibiotic treatment regimens, with no statistically significant differences observed (14). The study population of the above-mentioned meta-analysis exclusively comprised patients diagnosed with uncomplicated GNB-BSI. A recently published randomized controlled trial (RCT), the largest of its kind to date, evaluated the efficacy and safety of 7-day versus 14-day antibiotic regimens for BSI management (15). The results demonstrated that a 7-day antibiotic regimen was non-inferior to a 14-day regimen in patients with BSI. This study contributes significant, high-quality evidence to inform the selection of appropriate antibiotic treatment durations for the management of BSI. In light of these findings, we performed an updated systematic review and meta-analysis specifically focusing on patients with uncomplicated BSI. This study comparatively evaluated the clinical outcomes of 7-day versus 14-day antibiotic regimens, with dual objectives: (1) to assess efficacy (all-cause mortality, 90-day mortality) and (2) to assess safety profiles (relapsed bacteremia, adverse events, etc.). Our findings provide contemporary, evidence-based guidance for antimicrobial stewardship in uncomplicated BSI management.

## Methods

### Protocol and guidance

This systematic review and meta-analysis was conducted in accordance with the Preferred Reporting Items for Systematic Reviews and Meta Analysis (PRISMA) guidelines. Additionally, we have registered this study with the PROSPERO international prospective register of systematic reviews (Registration Number: CRD42024617359).

### Literature search strategy

Two researchers (WCM and DFL) independently conducted comprehensive searches of PubMed, Embase, Web of Science, and the Cochrane Library to identify relevant studies published from the inception of each database up to March 10th, 2025. No language restrictions were applied during the search process. Using the following terms and their combinations for literature search: “bloodstream OR bacteremia” And “antibiotic” And “duration OR days” And “randomized controlled trial.” After the initial search, the researchers thoroughly evaluated the full texts of all articles identified as potentially relevant. In cases where disagreements arose regarding the inclusion or exclusion of studies, the two authors engaged in discussions to reach a consensus. If a resolution could not be achieved through discussion, a third author (CYZ) was consulted to make the final decision. The comprehensive search strategies employed for each database, along with the corresponding search results, were presented in Supplementary file S1.

### Eligibility criteria

To determine whether the identified literature met the eligibility criteria, the two authors independently evaluated the titles, abstracts, and full texts of the studies. The inclusion of studies in this meta-analysis was based on the PICOS criteria, as outlined below:

1. Population: The study population comprised adult patients (aged ≥18 years) with microbiologically confirmed BSI, defined by at least one positive blood culture, and clinically diagnosed BSI based on standard criteria. Only uncomplicated BSI cases were included; patients with complicated BSI (e.g., BSI complicated by osteoarticular infections, central nervous system infections and endocarditis) were excluded. We intentionally maintained broad inclusion criteria regarding: Infection sources (all anatomical sites eligible); Pathogen spectrum (encompassing both Gram-negative and Gram-positive bacteria) to enhance the generalizability of our findings across real-world clinical settings.
2. Intervention: A 7-day antibiotic therapy.
3. Comparison: A 14-day antibiotic therapy.
4. Outcomes: Studies must report at least one of the following clinical outcomes: all-cause mortality, 90-day mortality, relapsed bacteremia, readmissions or prolongation of hospitalization, suppurative complications, emergence of resistance, length of stay in hospital, or adverse events, including acute kidney injury (AKI), *Clostridioides difficile* infection (CDI), diarrhea, and rash.
5. Study Design: Randomized controlled trials.

Regarding antibiotic regimens and administration methods, studies were included if they involved any choice of antibiotics (whether empirically determined or guided by drug sensitivity results) and any method of administration (oral or intravenous).

Exclusion Criteria: Studies were excluded if they met any of the following conditions:

1. The duration of treatment did not align with the prespecified treatment duration outlined in the study protocol;
2. The study lacked data on relevant clinical outcomes;
3. The study design included non-randomized controlled trials, semi-randomized trials, observational studies, systematic reviews, commentaries, editorials, narrative reviews, animal studies, or conference abstracts.

### Data extraction

Two researchers independently extracted relevant data from the eligible studies using a standardized data collection form. The following information was extracted from each study:

1. Basic Study Information: First author’s name, publication year, number of participating research centers, and the country where the study was conducted.
2. Baseline Patient Characteristics: Total number of patients included in the study.
3. Intervention Details: Whether the study used a 7-day antibiotic therapy or a 14-day antibiotic therapy.
4. Outcomes: Data on all-cause mortality, 90-day mortality, relapsed bacteremia, readmissions or prolongation of hospitalization, suppurative complications, emergence of resistance, length of stay in hospital, and adverse events. The definitions of key outcomes are provided in Supplementary file S2. AKI is defined in accordance with the Kidney Disease: Improving Global Outcomes (KDIGO) guidelines (16).

### Methodological quality assessment

The risk of bias for each included RCT was independently evaluated by two authors utilizing the risk of bias assessment tool outlined in the Cochrane Handbook for Systematic Reviews of Interventions. This comprehensive evaluation encompassed key domains such as random sequence generation, allocation concealment, blinding of participants and personnel, blinding of outcome assessment, incomplete outcome data, and selective reporting. A study was classified as having a low risk of bias only if all assessed domains were deemed to have a low risk of bias. Conversely, if any domain was identified as having a high risk of bias, the study was categorized as having a high overall risk of bias. To assess the overall certainty of the evidence, we employed the Grading of Recommendations Assessment, Development, and Evaluation (GRADE) methodology. Furthermore, we utilized the GRADEpro online software^1^ to construct a detailed GRADE evidence profile, ensuring a transparent and systematic presentation of the evidence quality.

### Statistical analysis

Data analysis was performed using Review Manager 5.4 software (Cochrane International Cooperation Organization) and STATA version 17.0 (StataCorp., College Station, TX). The significance level for the two-sided test was set at 0.05, and *p* < 0.05 was considered statistically significant. The effect statistics for continuous outcomes were analyzed using mean difference (MD) and standard deviation (SD), while dichotomous outcomes were analyzed using relative risk (RR) and 95% confidence interval (95% CI). Given the potential heterogeneity in both pathogen profiles and antibiotic treatment regimens across the included studies, a Dersimonian-Laird random-effects model was employed for the meta-analysis. Publication bias analysis was not performed due to the limited number of included studies.

To address potential random errors arising from limited sample sizes and multiple testing, we implemented Trial Sequential Analysis (TSA version 0.9.5.10 beta). The TSA computes the best statistics and appropriate significance boundaries for meta-analysis. In the TSA analysis, *α* = 0.05 (two-sided) and *β* = 0.20 were used to calculate the optimal sample size. Clear conclusions can be drawn when the cumulative Z curve crosses the TSA significance boundary, enters the invalid area, or reaches the optimal sample size. If the cumulative Z curve does not cross any boundaries, no clear conclusions can be drawn.

Our study population comprised patients with uncomplicated BSI, with particular focus on GNB-BSI. Among the four eligible studies identified through systematic review, only the trial by Daneman et al. (15) included patients with Gram-positive BSI, whereas GNB cases represented the predominant population (>70% of total cases). Given this distribution, we conducted subgroup analyses to evaluate all-cause mortality, and 90-day mortality. To ensure analytical precision for GNB-BSI, we subsequently performed dedicated subgroup analyses after excluding Gram-positive cases. This approach enabled specific assessment of treatment effects in Gram-negative bacteremia while maintaining statistical power.

## Results

### Literature retrieval

**Figure 1:**
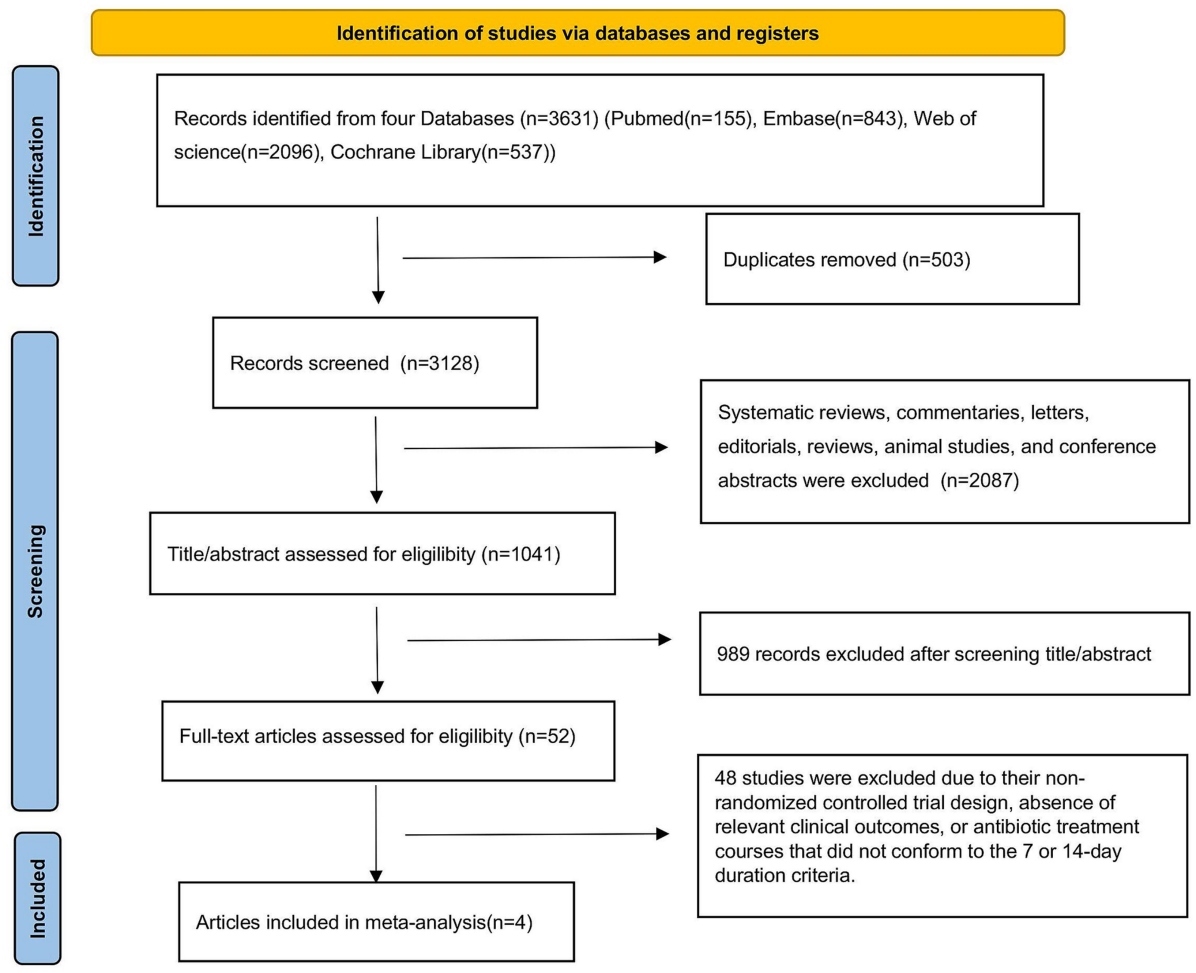
PRISMA flow diagram of the study selection process.

Our systematic literature search initially identified 3,631 potentially relevant articles across multiple databases, including 155 records from PubMed, 843 from EMBASE, 537 from the Cochrane Library, and 2,096 from Web of Science. Following the removal of duplicate entries and a comprehensive screening process involving title/abstract review and full-text evaluation, 4 RCTs (17–19) met the predefined inclusion criteria and were subsequently included in the final analysis (Figure 1).

### Methodological quality evaluation

**Figure 2:**
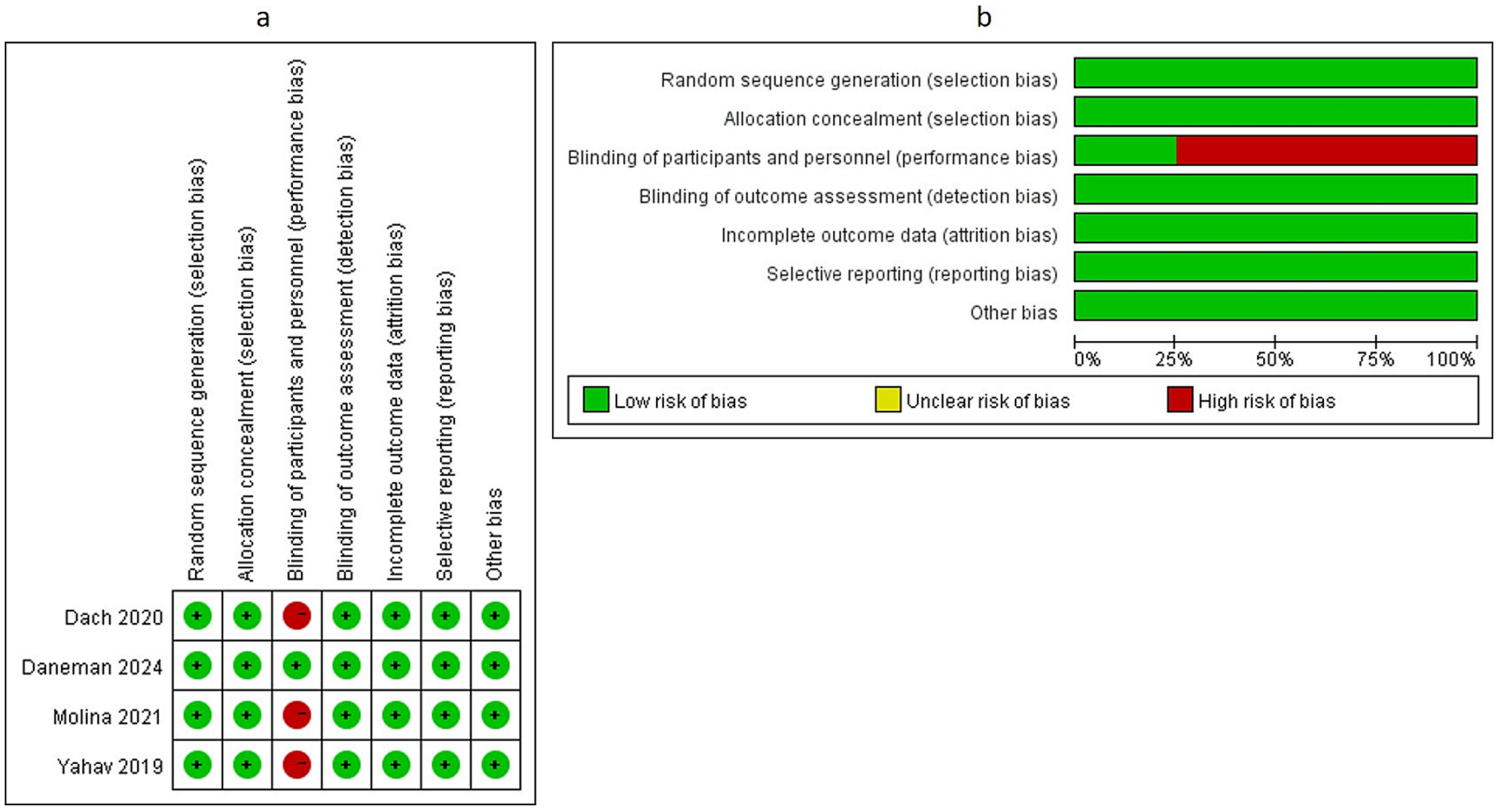
Methodological quality evaluation of included studies in this meta-analysis **(a)** risk-of-bias graph, **(b)**.risk-of-bias summary.

Among the four included RCTs, three studies (17–19) were identified as having a high risk of bias, primarily due to the absence of blinding procedures, which significantly increased the potential for risk of bias. The remaining RCT (15), which implemented appropriate blinding measures, was assessed as having a low risk of bias across all evaluated domains. A comprehensive visual representation of the methodological quality assessment, including the detailed risk of bias evaluation for each individual study across all specified domains, is presented in Figure 2.

### Basic characteristics of the included studies

Among the four multicenter RCTs included, two were conducted in Switzerland (18) and Spain (19), respectively. One study was a collaborative effort across Israel and Italy (17), while another spanned seven countries, including the United States and Canada (15). In terms of patient populations, two studies focused on adult patients diagnosed with GNB-BSI (17, 18). One study specifically enrolled patients with BSI caused by *Enterobacterales* (19), while the remaining study included patients with non-*Staphylococcus aureus* BSI (15). The urinary tract was consistently identified as the primary source of BSI across all included studies. The choice of antimicrobial agents, including both empirical treatment and targeted therapy guided by susceptibility testing, was made according to the clinical judgment of the treating physician. Detailed characteristics of the studies and the clinical outcomes available for analysis are summarized in Supplementary Table 1. Supplementary Table 2 summarized three key microbiological characteristics: (1) Main Source of bloodstream infections, (2) pathogen categories, and (3) Epidemiological Distribution of Predominant Pathogens (Supplementary Tables 1, 2 were included).

## Results of meta-analysis and TSA

### Primary outcome: all-cause mortality and 90-day mortality

#### All-cause mortality

**Figure 3:**
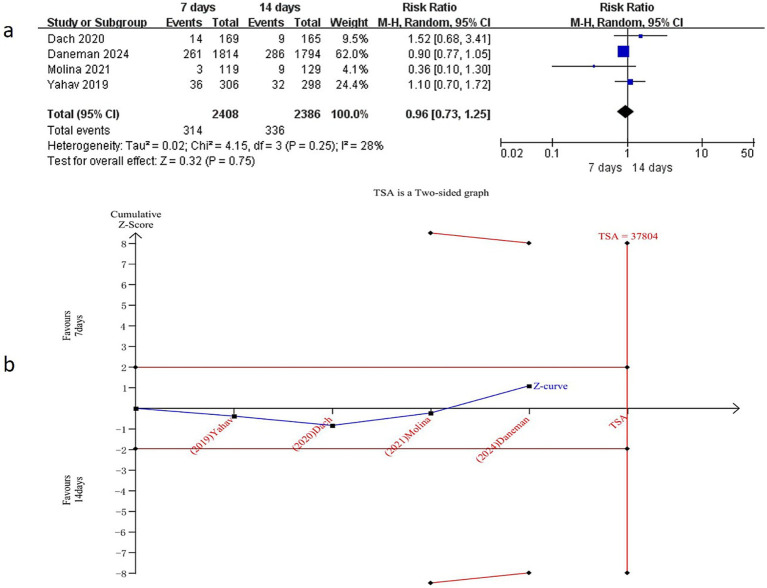
Comparison of 7-day versus 14-day antibiotic therapy on all-cause mortality **(a)** Forest plot of all-cause mortality, **(b)** Trial sequential analysis of 4 trials for all-cause mortality. The required information size for detecting an intervention effect was 37,804 patients.

All four studies (15, 17–19) reported data on all-cause mortality, encompassing a total of 4,794 patients (2,408 in the 7-day group and 2,386 in the 14-day group). Meta-analysis revealed no statistically significant difference in all-cause mortality between the 7-day and 14-day antibiotic regimens for BSI (RR = 0.96, 95% CI: 0.73–1.25, *p* = 0.75) (Figure 3a), with low heterogeneity observed (I^2^ = 28%, *p* = 0.25). Subgroup analysis restricted to Gram-negative BSI cases also showed no significant difference in all-cause mortality (RR = 0.92, 95% CI: 0.66–1.28, *p* = 0.62), with no substantial heterogeneity (I^2^ = 40%, *p* = 0.17), consistent with the initial findings (Supplementary Figure S1a).

#### 90-day mortality

**Figure 4:**
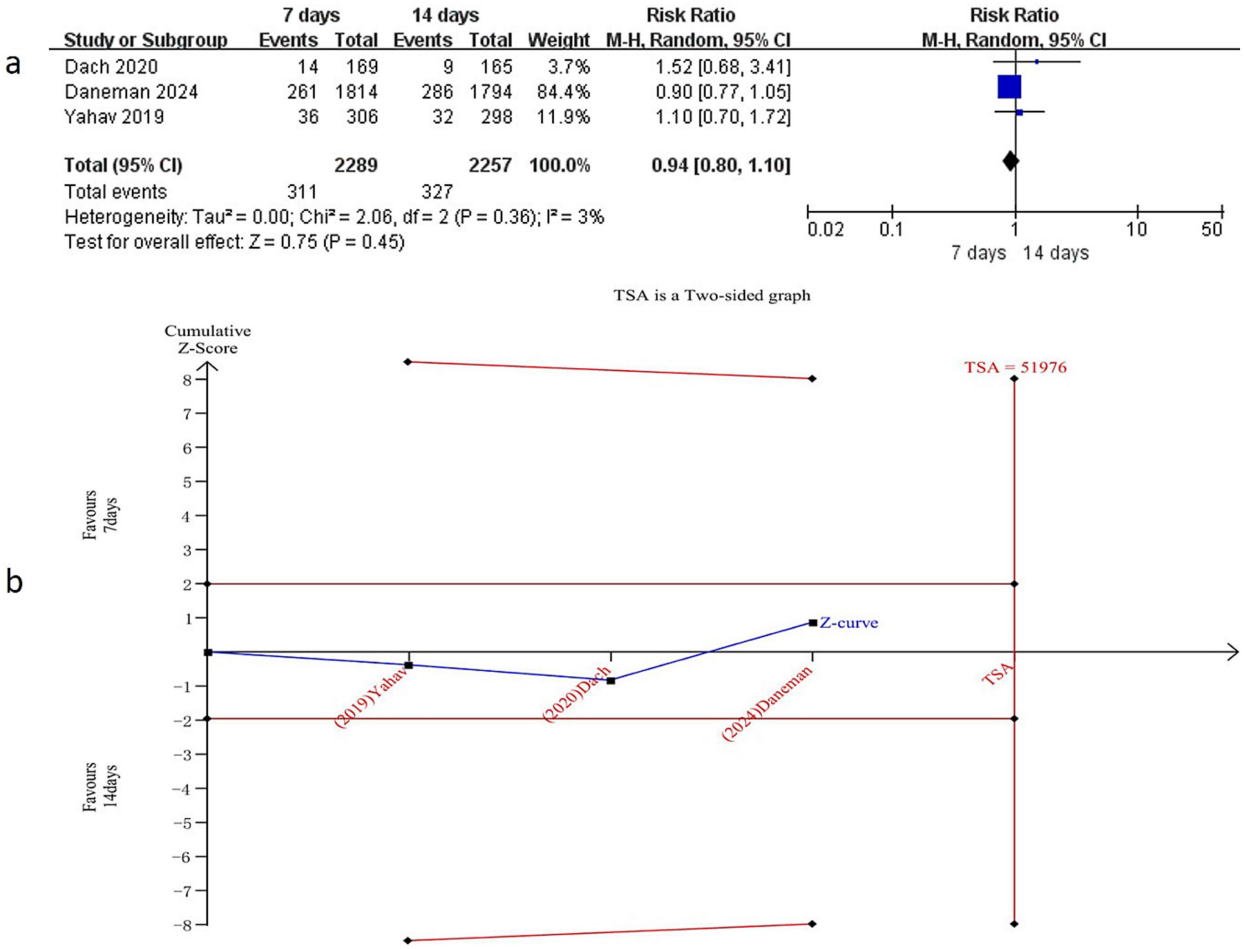
Comparison of 7-day versus 14-day antibiotic therapy on 90-day mortality **(a)** Forest plot of 90-day mortality, **(b)** Trial sequential analysis of 3 trials for 90-day mortality. The required information size for detecting an intervention effect was 51,976 patients.

Three studies (15, 17, 18) reported data on 90-day mortality, involving 4,546 patients (2,289 in the 7-day group and 2,257 in the 14-day group). No statistically significant difference in 90-day mortality was observed between the two groups (RR = 0.94, 95% CI: 0.80–1.10, *p* = 0.45) (Figure 4a), with no significant heterogeneity (I^2^ = 3%, *p* = 0.36). Subgroup analysis of patients with Gram-negative BSI similarly demonstrated no significant difference in 90-day mortality between the 7-day and 14-day antibiotic regimens (RR = 0.96, 95% CI: 0.71–1.29, *p* = 0.76) (Supplementary Figure S2a), with no significant heterogeneity (I^2^ = 38%, *p* = 0.20).

#### Trial sequential analysis (TSA)

To further validate the reliability of these findings and mitigate the risk of false-positive or false-negative results, TSA was performed. Based on mortality data extracted from the included RCTs, TSA was conducted for all-cause mortality, all-cause mortality in Gram-negative BSI, 90-day mortality, and 90-day mortality in Gram-negative BSI. The required information size (RIS) was calculated as 37,804, 51,976,12,310, and 14,884, respectively (Figures 3b, 4B, Supplementary Figures S1b, S2b). The cumulative Z-curve did not cross the conventional significance boundary or the TSA monitoring boundary, suggesting that the meta-analysis may have yielded false-negative results.

### Secondary outcomes

#### Relapsed bacteremia

**Figure 5:**
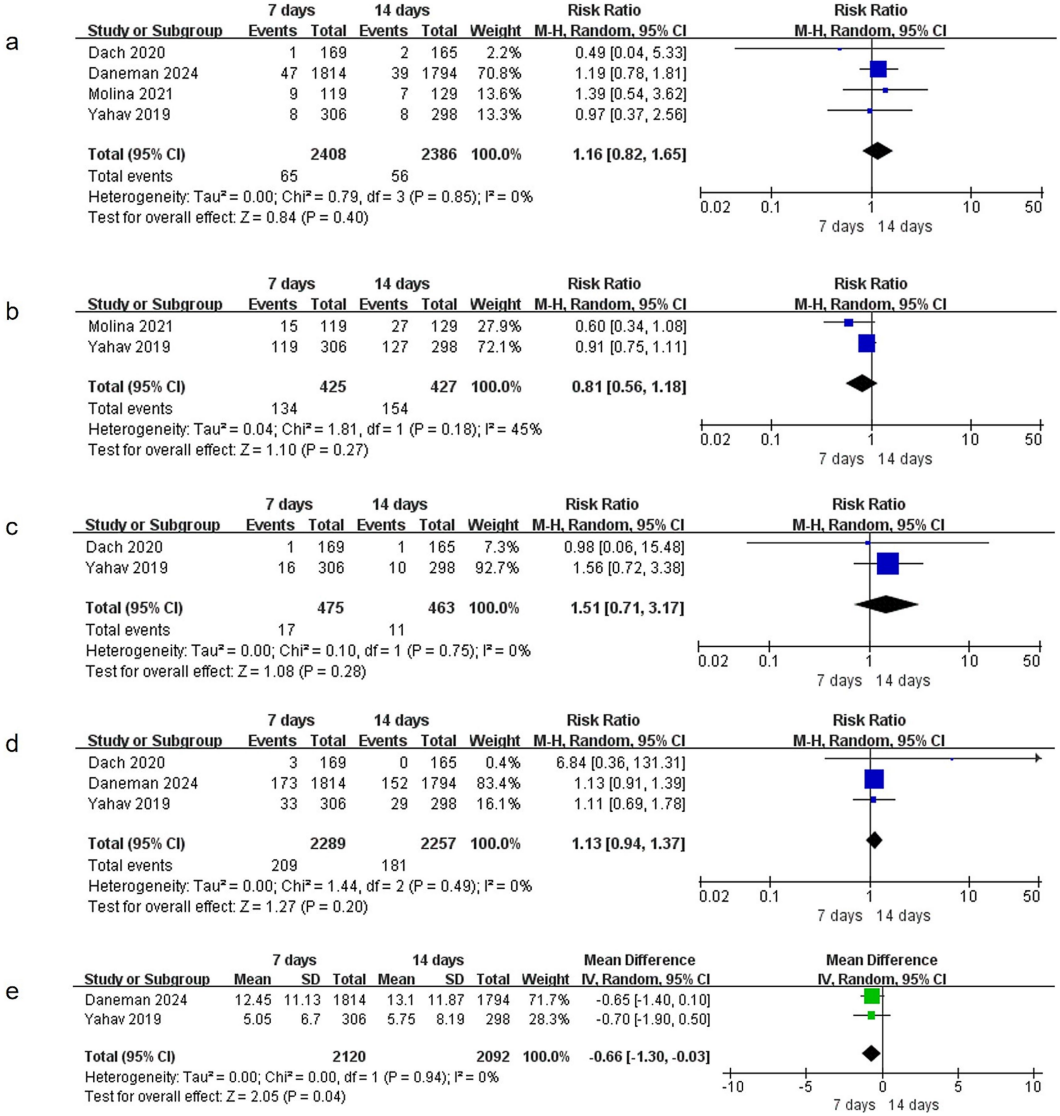
Comparison of 7-day versus 14-day antibiotic therapy on secondary outcomes **(a)** relapsed bacteremia, **(b)** readmissions or prolongation of hospitalization, **(c)** suppurative complications, **(d)** emergence of resistance, **(e)** Length of stay in hospital.

Four studies (15, 17–19) reported data on relapsed bacteremia, involving 4,794 patients (2,408 in the 7-day group and 2,386 in the 14-day group). No significant heterogeneity was observed (I^2^ = 0%, *p* = 0.85), and no statistically significant difference was found between the two groups (RR = 1.16, 95% CI: 0.82–1.65, *p* = 0.40) (Figure 5a).

#### Readmissions or hospitalization prolongation

Two studies (17, 19) provided data on readmissions or hospitalization prolongation, including 852 patients (425 in the 7-day group and 427 in the 14-day group). Heterogeneity was not significant (I^2^ = 45%, *p* = 0.18), and no statistically significant difference was observed between the groups (RR = 0.81, 95% CI: 0.56–1.18, *p* = 0.27) (Figure 5b).

#### Suppurative complications

Two studies (17, 18) reported data on suppurative complications, involving 938 patients (475 in the 7-day group and 463 in the 14-day group). No significant heterogeneity was detected (I^2^ = 0%, *p* = 0.75), and no statistically significant difference was found between the groups (RR = 1.51, 95% CI: 0.71–3.17, *p* = 0.28) (Figure 5c).

#### Emergence of resistance

Three studies (15, 17, 18) documented data on the emergence of resistance, enrolling 4,546 patients (2,289 in the 7-day group and 2,257 in the 14-day group). Heterogeneity was not significant (I^2^ = 0%, *p* = 0.49), and no statistically significant difference was observed between the groups (RR = 1.13, 95% CI: 0.94–1.37, *p* = 0.20) (Figure 5d).

#### Length of stay in hospital

Two studies (15, 17) reported data on the length of stay in hospital, involving 4,212 patients. No significant heterogeneity was observed (I^2^ = 0%, *p* = 0.94). The 7-day group was associated with a significantly shorter length of stay in hospital compared to the 14-day group (MD = −0.66, 95% CI: −1.30 to −0.03, *p* = 0.04) (Figure 5e).

### Adverse events

**Figure 6:**
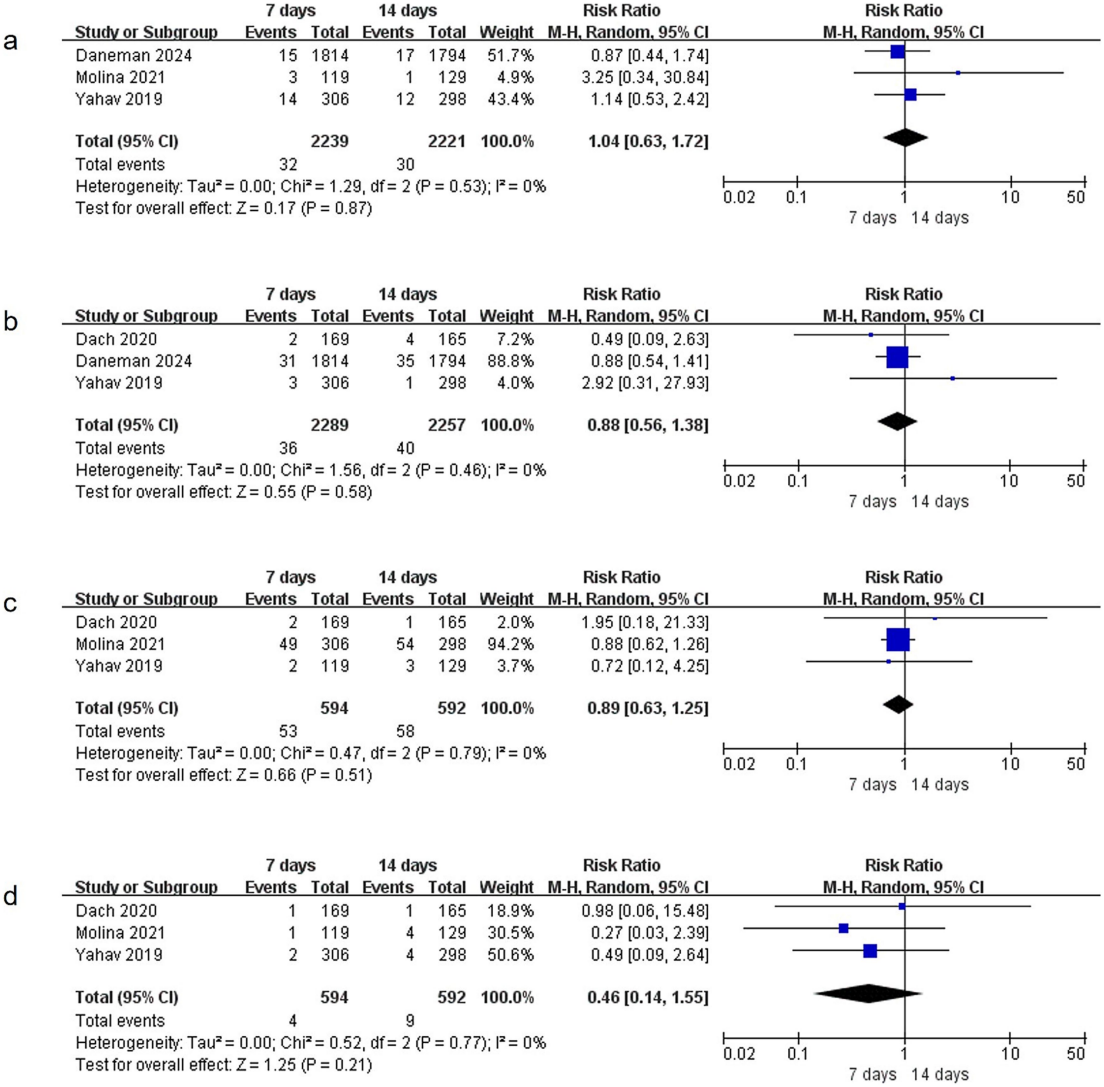
Comparison of 7-day versus 14-day antibiotic therapy on adverse events **(a)** AKI, **(b)** CDI, **(c)** diarrhea, **(d)** rash.

The meta-analysis evaluated four adverse events (AKI, CDI, diarrhea, and rash) across multiple studies. For AKI assessment, data from three studies (15, 17, 19) involving 4,460 patients showed no significant heterogeneity (I^2^ = 0%, *p* = 0.53) and revealed no between-group difference in incidence (RR = 1.04, 95% CI: 0.63–1.72, *p* = 0.87) (Figure 6a). Similarly, analysis of CDI data from three studies (15, 17, 18) (*n* = 4,546) demonstrated no significant heterogeneity (I^2^ = 0%, *p* = 0.46) and no association between antibiotic treatment duration and CDI incidence (RR = 0.88, 95% CI: 0.56–1.38, *p* = 0.58) (Figure 6b). Evaluation of diarrhea and rash outcomes from three studies (17–19) (n = 1,186) showed comparable incidence rates between groups, with RR = 0.89 (95% CI: 0.63–1.25, *p* = 0.51) (Figure 6c) for diarrhea and RR = 0.46 (95% CI: 0.14–1.55, *p* = 0.21) (Figure 6d) for rash, respectively. All analyses demonstrated consistent homogeneity across studies, with I^2^ values consistently at 0%.

### Sensitivity analysis

**Figure 7:**
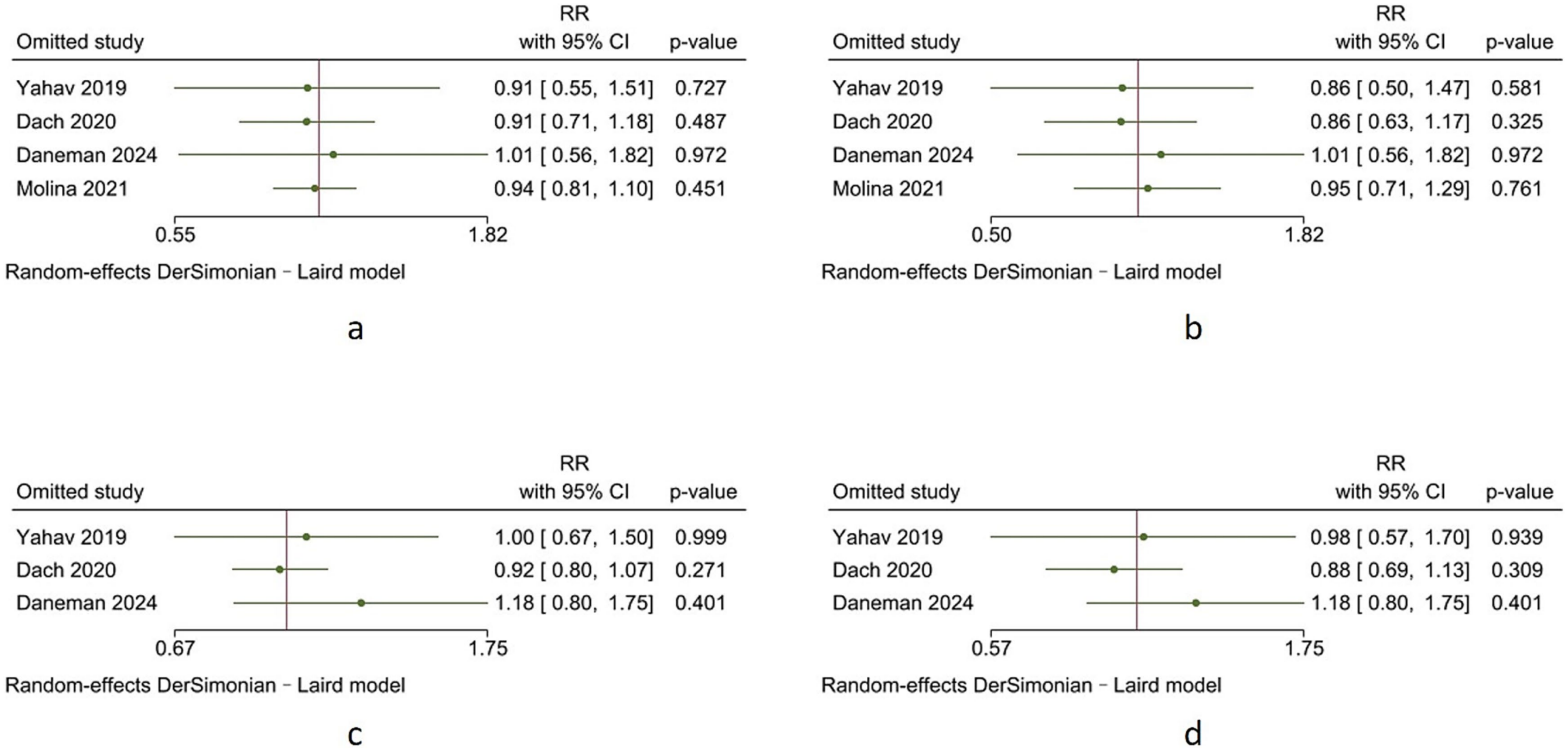
Sensitivity analysis **(a)** all-cause mortality, **(b)** all-cause mortality in patients with gram-negative bacterial BSI, **(c)** 90-day mortality, **(d)** 90-day mortality in patients with gram-negative bacterial BSI.

To evaluate the robustness of the meta-analysis results, we conducted a leave-one-out sensitivity analysis by systematically excluding each study one at a time and recalculating the pooled effect estimates. The sensitivity analysis demonstrated that the exclusion of any individual study did not significantly alter the overall effect estimates or the statistical significance of the findings (all *p*-values>0.05). This consistency across all iterations confirms the robustness and reliability of our meta-analysis results (Figure 7).

### GRADE certainty of the evidence

Upon evaluating the outcomes, the quality of evidence, as assessed by the GRADE criteria, was determined to range from low to high, with specific details provided in Supplementary Table 3.

## Discussion

This updated meta-analysis incorporated data from the largest RCT to date, demonstrating no significant difference in all-cause mortality or 90-day mortality between 7-day and 14-day antibiotic treatment regimens for uncomplicated non-*Staphylococcus aureus* BSI, particularly in cases of GNB-BSI. Trial sequential analysis revealed that the current sample size has not yet reached the required information size, suggesting the possibility of false-negative results. These findings underscore the need for further large-scale RCTs to validate the conclusions. Regarding secondary outcomes, the 7-day antibiotic regimen was associated with a shorter length of stay in hospital compared to the 14-day regimen. However, no significant differences were observed in relapsed bacteremia, readmissions or prolongation of hospitalization, suppurative complications, emergence of resistance. In terms of adverse events, both groups exhibited comparable incidences of AKI, CDI, diarrhea, and rash.

The optimal duration of antibiotic therapy remains a critical issue in clinical practice. Prolonged antibiotic use was associated with an increased risk of adverse effects (10, 20–23) and the emergence of antibiotic-resistant pathogens (24–26). Conversely, insufficient treatment duration may compromise therapeutic efficacy (27, 28) and lead to higher healthcare costs. Therefore, it is essential to balance minimizing the duration of antibiotic therapy to the shortest effective period while ensuring therapeutic efficacy, reducing adverse events, mitigating antimicrobial resistance, and alleviating the economic burden on healthcare systems.

The optimal duration of antibiotic therapy for BSI has been extensively investigated. A meta-analysis conducted by Li et al. (12) demonstrated that in patients with GNB-BSI, no significant differences were observed in 30-day mortality, 30-day recurrent bacteremia, 90-day mortality, or adverse event rates between short-course (6 to 11 days) and long-course (>10 days) antibiotic treatments. Similarly, another meta-analysis (13) focusing on *Enterobacteriaceae*-induced BSI, which included five studies involving 2,865 patients, reported no statistically significant differences in 30-day all-cause mortality, 90-day all-cause mortality, or clinical cure rates between short-course (≤10 days) and long-course (>10 days) antibiotic regimens. Furthermore, a meta-analysis restricted to RCTs (14) revealed that for uncomplicated GNB-BSI, a 7-day antibiotic course yielded comparable outcomes to a 14-day course in terms of mortality, recurrent bacteremia, hospital length of stay, infectious complications, drug resistance, and adverse events. Despite variations in the definitions and durations of antibiotic therapy across these studies, consistent findings demonstrate that the benefits of short-course antibiotic treatment for patients with bloodstream infections are not significantly inferior to those of long-course therapy.

In the current meta-analysis, three RCTs specifically enrolled patients diagnosed with GNB-BSI. An additional RCT (BALANCE trial) excluded patients who had a positive culture with a common contaminant (such as *coagulase-negative staphylococci*), had *Staphylococcus aureus* or *S. lugdunensis* bacteremia, bacteremia from rare organisms that required prolonged receipt of treatment, or fungemia. This RCT primarily focused on BSI caused by GNB, while also including cases involving Gram-positive cocci such as *enterococci.* Among the total cohort of 3,608 patients, 625 (17.3%) were identified as having Gram-positive bacterial infections (15). The study demonstrated that the 7-day antibiotic regimen was non-inferior to the 14-day antibiotic regimen, and consistent outcomes were observed in subgroup analyses following the exclusion of Gram-positive bacterial infections. These studies collectively demonstrated the feasibility and safety of 7-day antibiotic regimen in specific patient populations. At the same time, The BALANCE trial employed a stringent non-inferiority margin (*Δ* = 4%) for 90-day mortality, which was notably more conservative than conventional NI thresholds, thereby strengthening the validity of its non-inferiority conclusions. Although the BALANCE trial contributed a substantial proportion (76%) of the total sample size, sensitivity analysis demonstrated consistent results, indicating that its inclusion did not compromise the robustness of our findings. Although the trial TSA results suggested potential false-negative conclusions with the current data, incorporating the BALANCE trial significantly enhanced statistical power through sample size expansion, thereby strengthening the reliability of our findings.

While our meta-analysis demonstrated comparable all-cause and 90-day mortality rates between 7-day and 14-day antibiotic regimens, we found a wide range of all-cause mortality rates reported between different studies (2.5 to 15%). As illustrated by the 15% mortality rate in the BALANCE trial (7-day group) versus the 2.5% rate reported by Molina et al. This discrepancy primarily reflects differences in baseline disease severity: the BALANCE trial enrolled more critically ill patients, with a mean Sequential Organ Failure Assessment (SOFA) score of approximately 5, compared to mean scores of 2 in Yahav et al.’s trial and a quick SOFA (qSOFA) score of about 1 in von Dach et al.’s study.

However, it is crucial to identify which patients are appropriate for a 7-day antibiotic regimen and which patients require a 14-day or extended course of therapy. First, based on the shared characteristics identified across the four studies, a 7-day treatment course may be sufficient for uncomplicated GNB-BSI. Specifically, this applies to patients with positive blood cultures who exhibit no endocarditis, no indwelling medical devices, no metastatic infectious foci, and resolution of fever within 72 h of treatment initiation. In such cases, extending the treatment duration to 14 days is unlikely to yield additional clinical benefits. Second, for patients demonstrating early clinical improvement, such as normalization of body temperature and reduction in inflammatory markers, a 7-day treatment course may represent a safe and effective option. However, patients with complicated bloodstream infections, such as those involving concomitant endocarditis, osteomyelitis, or deep abscesses, typically require an extended treatment duration of 4–6 weeks to ensure complete eradication of the infection (7, 8). As demonstrated in the study by Babe et al. (29), patients with blood culture-positive infective endocarditis exhibit significantly higher risks of severe complications, including heart failure (HF) and septic shock, with mortality rates approaching 30%. In addition to surgical intervention when indicated, these findings support the clinical rationale for extended antibiotic therapy in this high-risk patient population. On the other hand, *Staphylococcus aureus* infections are often more invasive, and prolonged antibiotic therapy is generally recommended for *Staphylococcus aureus* BSI to minimize the risk of recurrence. Previous studies (27) have demonstrated that in Gram-positive bacterial bloodstream infections, particularly *Staphylococcus aureus* bacteremia, shorter antibiotic courses are associated with an increased risk of recurrence. Furthermore, immunocompromised patients, including organ transplant recipients, those with chemotherapy-induced neutropenia, or individuals on long-term immunosuppressive therapy, may require individualized treatment adjustments due to their heightened susceptibility to infections and more complex clinical conditions.

Studies investigating infections at various sites, beyond BSI, have also demonstrated comparable efficacy and safety between short-course and long-course antibiotic therapy (28, 30, 31). This evidence prompts the question: is less more? Minimizing antibiotic exposure while maintaining therapeutic efficacy is highly desirable, as it reduces the risk of adverse events and mitigates the development of antimicrobial resistance. However, it is critical to emphasize that timely source control is a cornerstone of effective infection management and plays a pivotal role in reducing the necessity for prolonged antibiotic therapy. For instance, in cases of catheter-related bloodstream infections, removing the central venous catheter and administering antibiotics for 7 days or fewer has been shown to be as safe and effective as longer treatment durations (32). Similarly, for BSI secondary to various infectious sources—such as urinary tract infections, localized abscesses, pneumonia, and intra-abdominal infections—source control may be more impactful than the duration of antibiotic therapy (33–35). These findings highlight the importance of a targeted approach to infection management, emphasizing source control alongside judicious antibiotic use.

We performed a comparative analysis to assess the efficacy and safety of two antibiotic treatment durations (7-day versus 14-day courses) for BSI. However, the study was limited by the unavailability of raw data, which precluded subgroup analyses based on critical factors such as pathogen type and infection source. Furthermore, trial sequential analysis indicated the potential for a false-negative outcome, necessitating cautious interpretation of the meta-analysis results. A recent individual patient data (IPD) meta-analysis (36) on this topic primarily demonstrated the non-inferiority of 7-day antibiotic therapy compared to 14-day treatment. Although the four included studies overlap with our analysis, our meta-analysis provides distinct methodological rigor and novel insights that enhance the current evidence base, our study remains a critical component of the evidence-based assessment in this field. Furthermore, our findings provide additional insights by highlighting potential risks of false-negative conclusions from alternative analytical perspectives. Moving forward, the ongoing SHORTEN-2 trial (37), a large-scale RCT, aims to further evaluate the efficacy and safety of a 7-day versus a 14-day antibiotic regimen for *Pseudomonas aeruginosa* bloodstream infections. We will closely monitor the publication of its findings and promptly update our systematic review and meta-analysis to integrate these results. This will enhance the evidence base for determining the optimal duration of antibiotic therapy in BSI, ultimately supporting improved clinical decision-making and patient outcomes.

We systematically evaluated the efficacy and safety of a 7-day antibiotic regimen compared to a 14-day regimen for the treatment of BSI. This study represents the most comprehensive meta-analysis to date, both in terms of sample size and the number of RCTs included. Furthermore, trial sequential analysis was conducted to validate the robustness of our findings. Nevertheless, several limitations should be acknowledged. First, significant heterogeneity was observed in the inclusion and exclusion criteria across the included studies. For example, two studies enrolled patients with mixed Gram-negative bacterial infections, one study focused exclusively on *Enterobacteriaceae* infections, and another included patients with BSI caused by a wide range of pathogens, including Gram-positive bacteria. Second, variations in antibiotic prescriptions, particularly in empirical treatment regimens, may have introduced considerable variability, potentially affecting the reliability of the results. Third, the lack of subgroup analyses based on pathogen type, antibiotic resistance profiles, and specific infection sources limits our ability to evaluate the impact of these factors on outcomes. Fourth, although TSA was performed to assess the reliability of our conclusions, the results suggest a potential risk of Type II errors (false negatives), which may undermine the robustness of the meta-analysis findings. And these findings underscore the critical need for additional large-scale RCTs to robustly validate these conclusions. Finally, it should be emphasized that our study specifically evaluated patients with uncomplicated GNB-BSI. While our findings provide important insights for GN-BSI management, their generalizability to bloodstream infections caused by other pathogens (e.g., Gram-positive bacteria or fungi) may be limited.

## Conclusion

Our systematic review and meta-analysis demonstrated that, for patients with BSI, a 7-day antibiotic regimen was associated with a shorter length of stay in hospital compared to a 14-day regimen. However, no significant differences were observed in mortality rates, relapsed bacteremia, readmissions or prolongation of hospitalization, suppurative complications, or the emergence of resistance. Similarly, both regimens showed comparable results in terms of adverse events. Trial sequential analysis suggested that the current findings may be subject to false-negative conclusions. Therefore, further high-quality RCTs with larger sample sizes are warranted to comprehensively evaluate the efficacy, safety, development of antibiotic resistance, and cost-effectiveness of 7-day versus 14-day regimens. Moreover, further high-quality studies are needed to explore the effects of varying antibiotic durations on different pathogens and to address the limitations identified in this analysis.

## Data Availability

The original contributions presented in the study are included in the article/Supplementary material, further inquiries can be directed to the corresponding author.

## Footnotes

^1^ https://www.gradepro.org/

## Supplementary material

SUPPLEMENTARY TABLE 1 The detailed characteristics of the included studies.

SUPPLEMENTARY TABLE 2 Key microbiological characteristics included in the study.

SUPPLEMENTARY TABLE 3 Grades of recommendation, assessment, development and evaluation summary of findings table.

SUPPLEMENTARY FILE S1 Literature search history and search results.

SUPPLEMENTARY FILE S2 Definitions of key outcomes.

SUPPLEMENTARY File S3 PRISMA checklist.

SUPPLEMENTARY FIGURE S1 Comparison of 7-day versus 14-day antibiotic therapy on all-cause mortality in patients with gram-negative bacterial BSI. **(a)** Forest plot of all-cause mortality in patients with gram-negative bacterial BSI. **(b)** Trial sequential analysis of 4 trials for all-cause mortality in patients with gram-negative bacterial BSI. The required information size for detecting an intervention effect was 12,310 patients.

SUPPLEMENTARY FIGURE S2 Comparison of 7-day versus 14-day antibiotic therapy on 90-day mortality in patients with gram-negative bacterial BSI. **(a)** Forest plot of 90-day mortality in patients with gram-negative bacterial BSI. **(b)** Trial sequential analysis of 3 trials for 90-day mortality in patients with gram-negative bacterial BSI. The required information size for detecting an intervention effect was 14,884 patients.

## Glossary

BSI: Bloodstream infections
RCTs: Randomized controlled trials
TSA: Trial Sequential Analysis
AKI: Acute kidney injury
CDI: Clostridioides difficile infection
GNB: Gram-negative bacteria
MRSA: *methicillin-resistant Staphylococcus aureus*
MDR-GNB: multidrug-resistant Gram-negative bacteria
IDSA: Infectious Diseases Society of America
SEIMC: The Spanish Society of Infectious Diseases and Clinical Microbiology
CRBSI: Catheter-related bloodstream infections
PRISMA: Preferred Reporting Items for Systematic Reviews and Meta Analysis
MeSH: Medical subject heading
SCr: serum creatinine
GRADE: Grading of Recommendations Assessment, Development, and Evaluation
RR: Relative risk
MD: Mean deviation
SD: Standard deviation
CI: Confidence interval
GNB-BSI: Gram-negative bacterial bloodstream infections
RIS: Required information size
SE: standard error
CVC: central venous catheter
HF: heart failure
SOFA: Sequential Organ Failure Assessment
qSOFA: Quick Sequential Organ Failure Assessment
