# Supplementary material for: Antibiotic treatment for 7 days versus 14 days in patients with uncomplicated bloodstream infections: a Systematic review and meta-analysis of randomized controlled trials and trial sequential analysis
Source: Front Med (Lausanne). 2025 Aug 4;12:1617328. doi: 10.3389/fmed.2025.1617328 (PMC12360037; doi:10.3389/fmed.2025.1617328)
Supplement: SUPPLEMENTARY TABLE 1 — The detailed characteristics of the included studies. [file Table_1.docx]

| Trial | Location | Study design | Patient | Inclusion criteria | Exclusion criteria | Non-inferiority Margin | No.of patients | Intervention | All-cause mortality | 28-day mortality | 30-day mortality | 60-day mortality | 90-day mortality | Emergence of resistance to study antibiotic | Length of stay in hospital | Relapsed bacteremia | Distant complication | Readmissions or prolongation of hospitalization | Suppurative complication | AKI | CDI | Diarrhea | Rash |
| --- | --- | --- | --- | --- | --- | --- | --- | --- | --- | --- | --- | --- | --- | --- | --- | --- | --- | --- | --- | --- | --- | --- | --- |
| Yahav 2019 | Israel and Italy | Multi-center Study;  RCT | Patients with Gram-negative bacteremia | Adults with growth of Gram-negative bacteria in one or more blood cultures, hemodynamically stable and afebrile for at least 48 h. | Sources of infection requiring prolonged treatment, fever or hemodynamic instability in the 48 h prior to randomization, uncontrolled focus of infection, polymicrobial growth involving Gram-positive bacteria, specific pathogens (*Brucella, Salmonella*), or specific immunosuppression (*human immunodeficiency virus*, neutropenia). | Δ <10%  all-cause mortality,  clinical failure, development of new clinically or microbiologically documented infection. | 306 | 7-day antibiotic therapy | 36/306 | 15/306 |  |  | 36/306 | 33/306 | IQR  4(1-10)days | 8/306 | 2/306 | 119/306 | 16/306 | 14/306 | 3/306 | 49/306 | 2/306 |
|  |  |  |  |  |  |  | 298 | 14-day  antibiotic therapy | 32/298 | 13/298 |  |  | 32/298 | 29/298 | IQR  4(1-12)days | 8/298 | 1/298 | 127/298 | 10/298 | 12/298 | 1/298 | 54/298 | 4/298 |
| Dach 2020 | Switzerland | Multi-center Study;  RCT | Patients with Gram-negative bacteremia | Adults with growth of Gram-negative fermenters in at least one blood culture and treatment with a microbiologically efficacious antibiotic. | Fever or hemodynamic instability in the 24 h prior to recruitment, severe immunosuppression, bacteremia with nonfermenting bacilli or polymicrobial, gram-positive growth, recurrent bacteremia, or complicated Infections. | The primary analysis used a 1-sided 97.5% CI for noninferiority. | 169 | 7-day antibiotic therapy | 14/169 |  | 6/169 | 11/169 | 14/169 | 3/169 |  | 1/169 | 0/169 |  | 1/169 |  | 2/169 | 2/169 | 1/169 |
|  |  |  |  |  |  |  | 165 | 14-day  antibiotic therapy | 9/165 |  | 4/165 | 8/165 | 9/165 | 0/165 |  | 2/165 | 0/165 |  | 1/165 |  | 4/165 | 1/165 | 1/165 |
| Molina 2021 | Spain | Multi-center Study;  RCT | Bacteremia caused by *Enterobacterales* | Adults with a diagnosis of *enterobacterales* bloodstream infections with appropriate source control. | Pregnancy, noncontrolled source of infection and no expectation of being controlled in the subsequent 24 h, patients undergoing chemotherapy with neutropenia <500 cells/mm3 expected for more than 7 days, infections requiring prolonged antibiotic treatment, infections caused by a carbapenemase producing member of the Enterobacterales, polymicrobial bacteraemia, and expectation of survival <48 h. | one-sided 97.5%CI for the difference between treatments in the proportion of patients were computed with the Newcombe–Wilson score method. | 119 | 7-day antibiotic therapy | 3/119 |  |  |  |  |  |  | 9/119 |  | 15/119 |  | 3/119 |  | 2/119 | 1/119 |
|  |  |  |  |  |  |  | 129 | 14-day  antibiotic therapy | 9/129 |  |  |  |  |  |  | 7/129 |  | 27/129 |  | 1/129 |  | 3/129 | 4/129 |
| Daneman 2024 | 7 countries including the USA, Canada, etc | Multi-center Study;  RCT | Patients with bloodstream infections | Patients were eligible for enrollment if they were admitted to a participating hospital at the time a blood culture was reported as positive with a pathogenic bacterium. | We excluded patients who had been previously enrolled in the trial, were severely immunocompromised (i.e., had neutropenia or were receiving immunosuppressive treatment after solid-organ transplantation or hematopoietic stem-cell transplantation), had prosthetic heart valves or endovascular grafts, had a documented or suspected infectious syndrome for which prolonged treatment was necessary (e.g., endocarditis, osteomyelitis, septic arthritis, undrained abscess, or unremoved prosthetic-associated infection), had a positive culture with a common contaminant (such as *coagulase-negative staphylococci*), had *Staphylococcus aureus* or*S. lugdunensis* bacteremia, bacteremia from rare organisms that required prolonged receipt of treatment, or fungemia. | Δ <4%  90-day mortality | 1814 | 7-day antibiotic therapy | 261/1814  166/1292（GNB） |  |  |  | 261/1814 | 173/1814 | IQR 10(6-21)days | 47/1814 |  |  |  | 15/1814 | 31/1814 |  |  |
|  |  |  |  |  |  |  | 1794 | 14-day  antibiotic therapy | 286/1794  197/1255（GNB） |  |  |  | 286/1794 | 152/1794 | IQR 11(6-22)days | 39/1794 |  |  |  | 17/1794 | 35/1794 |  |  |

Table 1 The detailed characteristics of the included studies

RCT, Randomized controlled trial; USA, the United States of America; IQR, interquartile range; GNB, Gram-negative bacteria; AKI, Acute kidney injury; CDI, *Clostridioides difficile* infection;
