# Supplementary material for: Antibiotic treatment for 7 days versus 14 days in patients with uncomplicated bloodstream infections: a Systematic review and meta-analysis of randomized controlled trials and trial sequential analysis
Source: Front Med (Lausanne). 2025 Aug 4;12:1617328. doi: 10.3389/fmed.2025.1617328 (PMC12360037; doi:10.3389/fmed.2025.1617328)
Supplement: SUPPLEMENTARY TABLE 2 — Key microbiological characteristics included in the study. [file Table_2.docx]

| **Study** | **Yahav 2019** | | **Dach 2020** | | **Molina 2021** | | **Daneman 2024** | |
| --- | --- | --- | --- | --- | --- | --- | --- | --- |
| Group | 7-day group | 14-day group | 7-day group | 14-day group | 7-day group | 14-day group | 7-day group | 14-day group |
| No.of patients | 306 | 298 | 169 | 165 | 119 | 129 | 1814 | 1794 |
| **Main Source of bloodstream infections** | | | | | | | | |
| Urinary tract (%) | 212（69.3） | 199（66.8） | 107（63.0） | 117（71.0） | 70/118（59.3） | 66/129（51.2） | 757（41.7） | 766（42.7） |
| Intraabdominal (%) | 37（12.1） | 34（11.4） | 37（22.0） | 20（12.0） | 16/118（13.6） | 18/129（14.0） | 337（18.6） | 342（19.1） |
| Lungs (%) | 14（4.6） | 10（3.4） | 14（8.0） | 16（10.0） | 3/118（2.5） | 12/129（9.3） | 229（12.6） | 240（13.4） |
| Vascular catheter/endovascular device (%) | 15（4.9） | 23（7.7） | 5（3.0） | 5（3.0） | 14/118（11.9） | 16/129（12.4） | 116（6.4） | 113（6.3） |
| Skin and soft tissue (%) | 5（1.6） | 4（1.3） | - | - | - | - | 104（5.7） | 83（4.6） |
| **Pathogen categories** | | | | | | | | |
| Gram-negative bacteria (%) | 306（100） | 298（100） | 169（100） | 165（100） | 119（100) | 129(100) | 1299(71.6) | 1263(70.4) |
| Gram-positive bacteria (%) | - | - | - | - | - | - | 323(17.8) | 302(16.8) |
| Polymicrobial (%) | - | - | - | - | - | - | 192(10.6) | 229(12.8) |
| **Epidemiological Distribution of Predominant Pathogens** | | | | | | | | |
| Escherichia coli (%) | 186(60.8) | 194(65.1) | 123(73.0) | 124(75.0) | 76(66.4) | 79(61.2) | 805(44.4) | 777(44.3) |
| Klebsiella pneumoniae (%) | 47(15.3) | 33(11.1) | 35(21.0) | 26(16.0) | 23(19.5) | 23(17.8) | 273(15.0) | 279(15.6) |
| Pseudomonas species (%) | 28(9.2) | 20(6.7) | - | - | - | - | 80(4.4) | 90(5.0) |
| Enterococcus genus (%) | - | - | - | - | - | - | 119(6.6) | 131(7.3) |
| Enterobacter species (%) | - | - | 3(2.0) | 1(1.0) | 11(9.2) | 15(11.6)) | 80(4.4) | 77(4.3) |
| Proteus species (%) | - | - | 7(4.0) | 6(4.0) | - | - | 58(3.2) | 75(4.2) |
| Serratia species (%) | - | - | - | - | 3(2.5) | 4(3.1) | 38(2.1) | 48(2.7) |
| Acinetobacter species (%) | 2(0.7) | 4(1.3) | - | - | - | - | 24(1.3) | 16(0.9) |
| Streptococcus pneumoniae (%) | - | - | - | - | - | - | 86(4.7) | 78(4.3) |
| Coagulase-negative staphylococci (%) | - | - | - | - | - | - | 81(4.5) | 93(5.2) |
| S. agalacticae (%) | - | - | - | - | - | - | 40(2.2) | 3.5(2.0) |
