# Supplementary material for: Antibiotic treatment for 7 days versus 14 days in patients with uncomplicated bloodstream infections: a Systematic review and meta-analysis of randomized controlled trials and trial sequential analysis
Source: Front Med (Lausanne). 2025 Aug 4;12:1617328. doi: 10.3389/fmed.2025.1617328 (PMC12360037; doi:10.3389/fmed.2025.1617328)
Supplement: SUPPLEMENTARY TABLE 3 — Grades of recommendation, assessment, development and evaluation summary of findings table. [file Table_3.docx]

Table 2. Grades of recommendation, assessment, development and evaluation summary of findings table.

| **Certainty assessment** | | | | | | | **№ of patients** | | **Effect** | | **Certainty** | **Importance** |
| --- | --- | --- | --- | --- | --- | --- | --- | --- | --- | --- | --- | --- |
| **№ of studies** | **Study design** | **Risk of bias** | **Inconsistency** | **Indirectness** | **Imprecision** | **Other considerations** | **7 days** | **14 days** | **Relative (95% CI)** | **Absolute (95% CI)** |  |  |
| **All-cause mortality** | | | | | | | | | | | | |
| 4 | randomised trials | serious | not serious | serious | not serious | all plausible residual confounding would reduce the demonstrated effect | 314/2408 (13.0%) | 336/2386 (14.1%) | **RR 0.96** (0.73 to 1.25) | **6 fewer per 1,000** (from 38 fewer to 35 more) | ⨁⨁⨁◯ Moderate | CRITICAL |
| **90-day mortality** | | | | | | | | | | | | |
| 3 | randomised trials | serious | not serious | serious | not serious | all plausible residual confounding would reduce the demonstrated effect | 311/2289 (13.6%) | 327/2257 (14.5%) | **RR 0.94** (0.80 to 1.10) | **9 fewer per 1,000** (from 29 fewer to 14 more) | ⨁⨁⨁◯ Moderate | IMPORTANT |
| **Relapsed bacteremia** | | | | | | | | | | | | |
| 4 | randomised trials | serious | not serious | serious | not serious | all plausible residual confounding would reduce the demonstrated effect | 65/2408 (2.7%) | 56/2386 (2.3%) | **RR 1.16** (0.82 to 1.65) | **4 more per 1,000** (from 4 fewer to 15 more) | ⨁⨁⨁◯ Moderate | IMPORTANT |
| **Readmissions** | | | | | | | | | | | | |
| 2 | randomised trials | serious | not serious | serious | serious | all plausible residual confounding would reduce the demonstrated effect | 134/425 (31.5%) | 154/427 (36.1%) | **RR 0.81** (0.56 to 1.18) | **69 fewer per 1,000** (from 159 fewer to 65 more) | ⨁⨁◯◯ Low | IMPORTANT |
| **Suppurative complication** | | | | | | | | | | | | |
| 2 | randomised trials | serious | not serious | serious | serious | all plausible residual confounding would reduce the demonstrated effect | 17/475 (3.6%) | 11/463 (2.4%) | **RR 1.51** (0.71 to 3.17) | **12 more per 1,000** (from 7 fewer to 52 more) | ⨁⨁◯◯ Low | IMPORTANT |
| **Emergence of resistance to study** | | | | | | | | | | | | |
| 3 | randomised trials | serious | not serious | serious | not serious | all plausible residual confounding would reduce the demonstrated effect | 209/2289 (9.1%) | 181/2257 (8.0%) | **RR 1.13** (0.94 to 1.37) | **10 more per 1,000** (from 5 fewer to 30 more) | ⨁⨁⨁◯ Moderate | IMPORTANT |
| **AKI** | | | | | | | | | | | | |
| 3 | randomised trials | serious | not serious | serious | not serious | all plausible residual confounding would reduce the demonstrated effect | 32/2239 (1.4%) | 30/2221 (1.4%) | **RR 1.04** (0.63 to 1.72) | **1 more per 1,000** (from 5 fewer to 10 more) | ⨁⨁⨁◯ Moderate | IMPORTANT |
| **CDI** | | | | | | | | | | | | |
| 3 | randomised trials | serious | not serious | serious | not serious | all plausible residual confounding would reduce the demonstrated effect | 36/2289 (1.6%) | 40/2257 (1.8%) | **RR 0.88** (0.56 to 1.38) | **2 fewer per 1,000** (from 8 fewer to 7 more) | ⨁⨁⨁◯ Moderate | IMPORTANT |
| **Diarrhea** | | | | | | | | | | | | |
| 3 | randomised trials | serious | not serious | serious | serious | all plausible residual confounding would reduce the demonstrated effect | 53/594 (8.9%) | 58/592 (9.8%) | **RR 0.89** (0.63 to 1.25) | **11 fewer per 1,000** (from 36 fewer to 24 more) | ⨁⨁◯◯ Low | IMPORTANT |
| **Rash** | | | | | | | | | | | | |
| 3 | randomised trials | serious | not serious | serious | very serious | all plausible residual confounding would reduce the demonstrated effect | 4/594 (0.7%) | 9/592 (1.5%) | **RR 0.46** (0.14 to 1.55) | **8 fewer per 1,000** (from 13 fewer to 8 more) | ⨁◯◯◯ Very low | IMPORTANT |
| **All-cause mortality (Gram-negative bacteria)** | | | | | | | | | | | | |
| 4 | randomised trials | serious | not serious | not serious | not serious | all plausible residual confounding would reduce the demonstrated effect | 219/1886 (11.6%) | 247/1847 (13.4%) | **RR 0.92** (0.66 to 1.28) | **11 fewer per 1,000** (from 45 fewer to 37 more) | ⨁⨁⨁⨁ High | CRITICAL |
| **90-day mortality (Gram-negative bacteria)** | | | | | | | | | | | | |
| 3 | randomised trials | serious | not serious | not serious | not serious | all plausible residual confounding would reduce the demonstrated effect | 216/1767 (12.2%) | 238/1718 (13.9%) | **RR 0.96** (0.71 to 1.29) | **6 fewer per 1,000** (from 40 fewer to 40 more) | ⨁⨁⨁⨁ High | CRITICAL |
| **length of stay in hospital** | | | | | | | | | | | | |
| 2 | randomised trials | serious | not serious | serious | not serious | all plausible residual confounding would reduce the demonstrated effect | 2120 | 2092 | - | MD **0.66 lower** (1.3 lower to 0.03 lower) | ⨁⨁⨁◯ Moderate | CRITICAL |

**CI:** confidence interval; **MD:** mean difference; **RR:** risk ratio
