# Supplementary material for: Antibiotic treatment for 7 days versus 14 days in patients with uncomplicated bloodstream infections: a Systematic review and meta-analysis of randomized controlled trials and trial sequential analysis
Source: Front Med (Lausanne). 2025 Aug 4;12:1617328. doi: 10.3389/fmed.2025.1617328 (PMC12360037; doi:10.3389/fmed.2025.1617328)
Supplement: SUPPLEMENTARY FILE S2 — Definitions of key outcomes. [file Data_Sheet_2.docx]

Definitions of Key Outcomes:

All-cause mortality: The data primarily include the 90-day mortality rate. However, in the study by Molina et al. (2021), follow-up was limited to 28 days after the cessation of antibiotic treatment. Due to inconsistencies in the duration of antibiotic treatment between the two groups, only all-cause mortality data were available for analysis.

Relapsed bacteremia: Defined as the recurrence of bacteremia caused by the same bacterial strain, occurring from the time of randomization until the end of the follow-up period.

Emergence of resistance: Defined as the development of resistance by the pathogenic bacteria to the antibiotics under investigation at any point during the study.

Suppurative complication: Defined as a localized purulent complication that was not present at the onset of the infection.

AKI: Defined as an increase in serum creatinine (SCr) of ≥0.3 mg/dL (≥26.5μmol/L) within 48 hours; or an increase in serum creatinine to ≥1.5 times the baseline level, known or presumed to have occurred within the past 7 days; or a urine output of <0.5 mL/kg/h for 6 hours.
