# Supplementary figures and images for: Antibiotic treatment for 7 days versus 14 days in patients with uncomplicated bloodstream infections: a Systematic review and meta-analysis of randomized controlled trials and trial sequential analysis
Source: Front Med (Lausanne). 2025 Aug 4;12:1617328. doi: 10.3389/fmed.2025.1617328 (PMC12360037; doi:10.3389/fmed.2025.1617328)

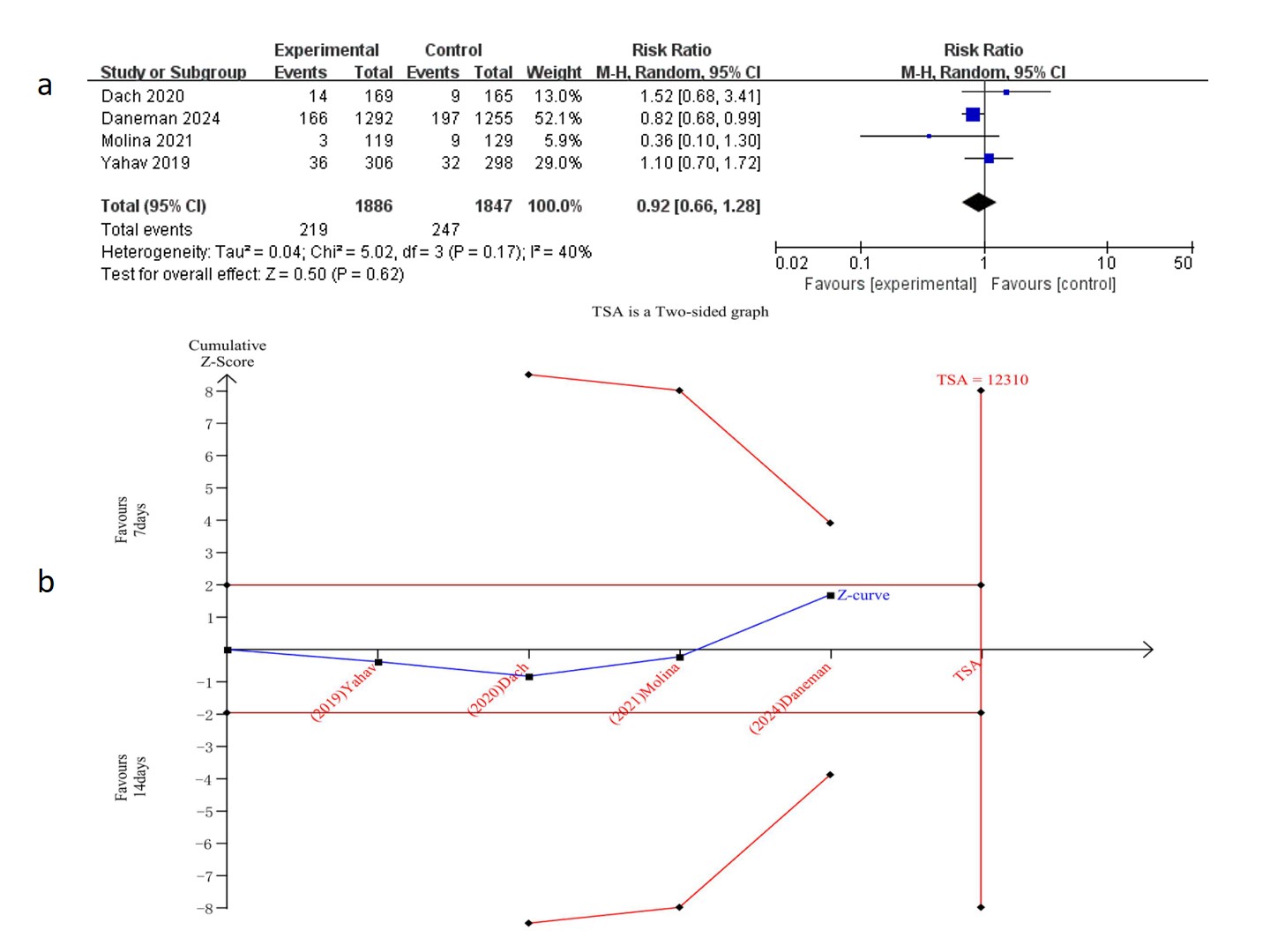

Supplement: SUPPLEMENTARY FIGURE S1 — Comparison of 7-day versus 14-day antibiotic therapy on all-cause mortality in patients with gram-negative bacterial BSI. (a) Forest plot of all-cause mortality in patients with gram-negative bacterial BSI. (b) Trial sequential analysis of 4 trials for all-cause mortality in patients with gram-negative bacterial BSI. The required information size for detecting an intervention effect was 12,310 patients. [file Image_1.JPEG]

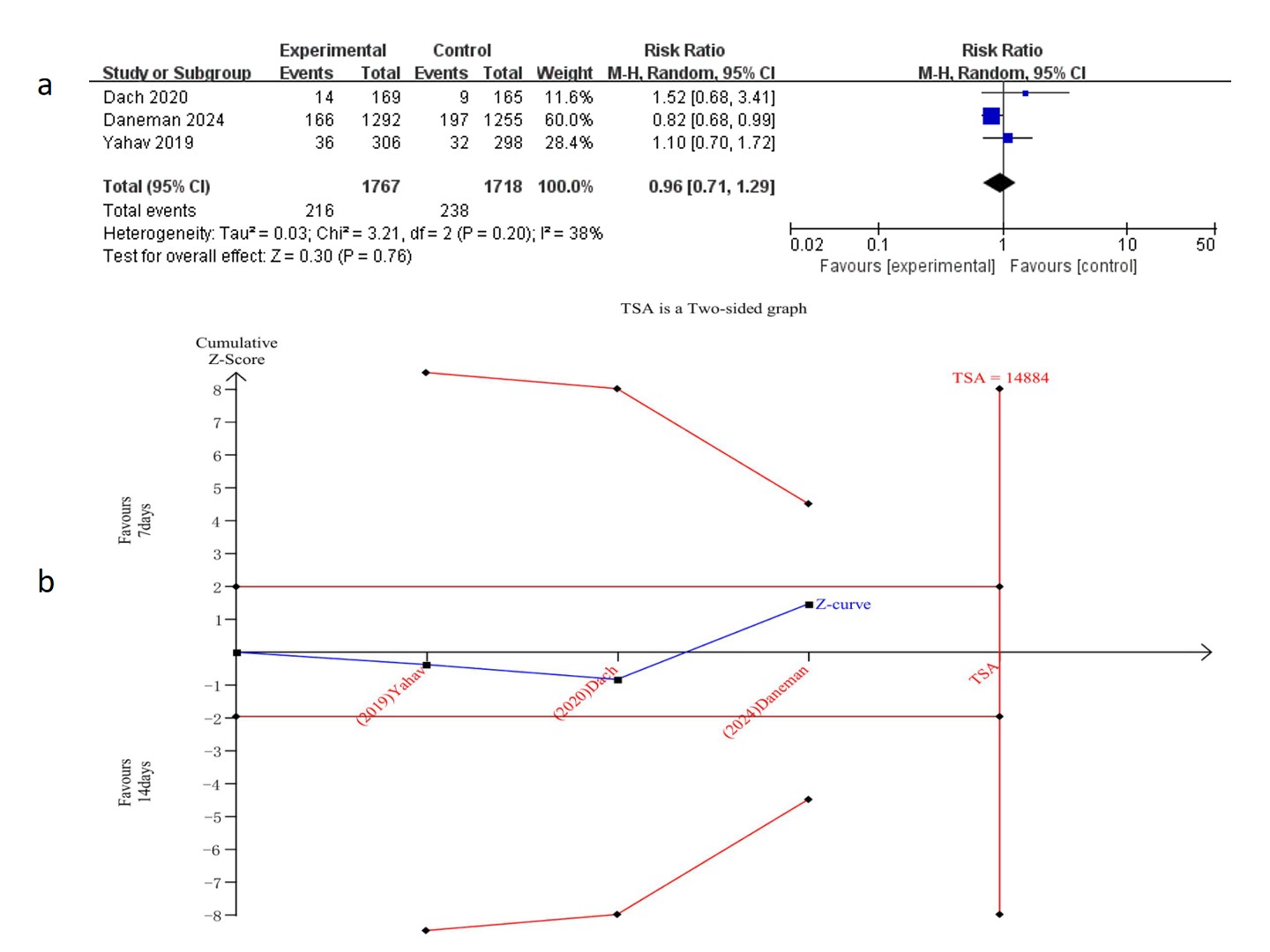

Supplement: SUPPLEMENTARY FIGURE S2 — Comparison of 7-day versus 14-day antibiotic therapy on 90-day mortality in patients with gram-negative bacterial BSI. (a) Forest plot of 90-day mortality in patients with gram-negative bacterial BSI. (b) Trial sequential analysis of 3 trials for 90-day mortality in patients with gram-negative bacterial BSI. The required information size for detecting an intervention effect was 14,884 patients. [file Image_2.JPEG]
